# Anatomical Determinants of STN Coordinate Shift in Idiopathic Parkinson's Disease DBS Surgery

**DOI:** 10.1111/cns.70307

**Published:** 2025-04-24

**Authors:** Ozan Hasimoglu, Tuba Özge Karaçoban, Taha Hanoglu, Nur Bahar Geylan, Ayca Altinkaya, Buruc Erkan, Lütfi Şinasi Postalci, Bekir Tugcu

**Affiliations:** ^1^ Neurosurgery Department, University of Health Sciences, Hamidiye Faculty of Medicine, Basaksehir Cam and Sakura City Hospital Istanbul Turkey; ^2^ Neurology Department, University of Health Sciences, Hamidiye Faculty of Medicine, Basaksehir Cam and Sakura City Hospital Istanbul Turkey

**Keywords:** anatomical variability, brain Volumetry, deep brain stimulation (DBS), idiopathic Parkinson's disease, subthalamic nucleus (STN)

## Abstract

**Objective:**

This study examines how anatomical variations influence the targeting coordinates of the subthalamic nucleus (STN) in patients with Idiopathic Parkinson's Disease (IPD) undergoing Deep Brain Stimulation (DBS), with the goal of enhancing targeting accuracy.

**Methods:**

A retrospective analysis was performed on 202 STNs from patients who received bilateral STN‐DBS surgery. Pre‐ and postoperative imaging data were used to determine accurate STN coordinates, while brain volume measurements, ventricle size, Evans Index, and AC –PC length were analyzed. Atrophy grading scales were also applied. Correlation and regression analyses assessed the relationship between the STN target location and all anatomical parameters on the *x*, *y*, and *z* axes.

**Results:**

Age showed a significant positive correlation with lateral STN coordinate shift on the *x*‐axis, with each additional year leading to a 0.046 mm shift. An increase in peripheral gray matter volume and a decrease in white matter volume were significantly associated with the lateral displacement of the STN. Total ventricle volume demonstrated a positive correlation with STN shift on both the *x*‐axis (0.0227 mm per cm^3^ increase) and *z*‐axis (0.0087 mm per cm^3^ increase). Significant correlations were also found for the Evans Index with lateral shift on the *x*‐axis and for AC‐PC length with vertical shifts.

**Conclusion:**

Anatomical factors, such as brain volume, ventricle size, Evans Index, AC‐PC length, and atrophy scores, significantly influence STN localization in PD patients undergoing DBS. Accounting for these shifts during surgical planning may improve electrode placement accuracy and enhance therapeutic outcomes, underscoring the importance of personalized targeting strategies.

## Graphical abstract

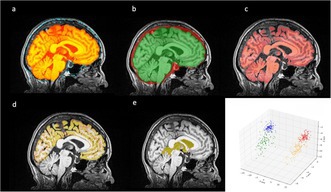

**Abbreviations**
AC‐PC: anterior commissure‐posterior commissure
BET: brain extraction tool
CT: computed tomography
DBS: deep brain stimulation
DTI: diffusion tensor imaging
FSL: FMRIB software library
GCA‐F: global cortical atrophy‐frontal
IPD: idiopathic Parkinson's disease
MCP: midcommissural point
MER: microelectrode recording
MNI: montreal neurological institute
MRI: magnetic resonance imaging
MTA: medial temporal atrophy (Scale)
PDQ‐39: Parkinson's disease questionnaire
STN: subthalamic nucleus
UPDRS: unified Parkinson's disease rating scale

## 1 Introduction

Parkinson's disease is a neurodegenerative syndrome involving multiple motor and non‐motor neural circuits in the basal ganglia [1]. Deep‐brain stimulation is a surgical technique in which electrodes are implanted in specific regions of the brain [2]. The Subthalamic Nucleus (STN) is the most commonly targeted site for stimulation in the treatment of idiopathic Parkinson disease (IPD) [3]. Precise placement of electrodes at the designated target in DBS therapy is crucial for the effectiveness of the treatment [4, 5, 6].

Targeting in DBS therapy can be achieved through direct or indirect methods. Indirect targeting relies on standardized coordinates derived from anatomical atlases. According to atlas‐based standards, the STN is typically located at coordinates relative to the anterior commissure –posterior commissure (AC‐PC) midpoint: *x*, ±12; *y*, −2/3; and *z*, −3/4 [4, 7]. The indirect targeting method does not account for individual anatomical differences, which may result in inaccurate electrode placement [8]. Certain structural variations and individual characteristics may contribute to these targeting inaccuracies. Consequently, various indirect targeting methods have occasionally been developed and tested [9].

Advancements in imaging techniques, particularly the use of high‐resolution Magnetic Resonance Imaging (MRI), have enabled direct visualization of structures like the STN, enabling more accurate and individualized targeting [4, 10, 11]. Additionally, atlas‐based automatic segmentation techniques have now become commonplace, allowing for highly precise visualization of nuclear positions [12]. Despite these advancements in imaging techniques and segmentation methods, achieving accurate STN coordinate predictions remains challenging. These difficulties persist even with the availability of high‐resolution imaging and atlas‐based tools, underscoring the need for continued refinement of targeting strategies [11].

Given these challenges, this study aims to examine the variability of STN targeting coordinates selected by clinicians and associated with successful outcomes during the planning process for STN‐DBS surgery in Parkinson's patients. Specifically, it analyzes how anatomical variations, such as atrophy and hydrocephalus, influence these coordinates. By quantifying these anatomical changes and their relationship with coordinate shifts, it aims to provide clinicians with actionable insights to improve and adapt anatomical targeting strategies.

## 2 Methods

### 2.1 Participant Selection

This study included patients diagnosed with IPD who underwent bilateral STN‐DBS performed by the same surgical team (BT, OH) between 2011 and 2024. Surgical decisions were made by a multidisciplinary movement disorders board, which consisted of a neurosurgeon, movement disorder neurologist, psychiatrist, neuropsychologist, speech analyst, and physiotherapist. All patients were evaluated based on objective criteria set by the board and were deemed eligible for DBS surgery following this assessment. Following the decision by the movement disorders board, each patient underwent a comprehensive clinical evaluation, including the Unified Parkinson's Disease Rating Scale (UPDRS), Parkinson's Disease Questionnaire (PDQ‐39), and a series of neuropsychological test batteries. All patients included in the study underwent these tests both before and after surgery. Patients who showed at least a 30% improvement in the UPDRS Part III (UPDRS‐III) scores under medication‐off conditions after stimulation did not experience intolerable side effects and continued to use their DBS systems actively were considered to have benefited from DBS and were included in the study. This criterion was employed because our study is not focused on clinical outcomes but aims to minimize potential bias in anatomical localization analyzes that could arise from including patients who did not benefit from DBS. Patients who did not derive benefit from DBS, as well as those with incomplete or inaccessible retrospective MRI data, were excluded from the study.

### 2.2 Planning and Surgical Procedure

Before surgery, all patients underwent high‐resolution MRI with either 1.5 or 3 Tesla, performed according to standard protocols 1–3 days before the operation. MRI sequences included T1, T2, and contrast‐enhanced T1 images, along with Diffusion Tensor Imaging (DTI) to map white matter tracts. These images were routinely processed and stored. Targeting was conducted using both direct and indirect methods. The dorsolateral region of the STN was designated as the optimal target for managing the motor symptoms of Idiopathic Parkinson's Disease. Target coordinates were calculated using the AC‐PC line (anterior commissure‐posterior commissure) as a reference, with the midpoint between the anterior and posterior commissures, known as the mid‐commissural point (MCP), set as the origin. Initial coordinates of *X*, ±12; *Y*, −2; and *Z*, −4 were entered for all patients, followed by adjustments toward the dorsolateral STN through direct visualization. Planning was executed using surgical navigation software, including Stealth and BrainLab Elements. In most cases, the BrainLab object manipulation module with automatic segmentation was employed as a supportive tool to confirm STN positioning, complementing the clinician's judgment based on direct visualization. The planning coordinates accepted as the reference for this study were recorded at this stage. STN coordinate shifts were analyzed relative to these planning coordinates to assess how anatomical variations influenced the spatial positioning of the STN during DBS surgery planning.

On the day of surgery, most patients underwent the procedure while awake. The stereotactic frame was affixed in the morning, and a 1 mm CT scan was performed with the frame to fuse with planning images, capturing stereotactic frame coordinates. All patients received microelectrode recording (MER) and macrostimulation. During the procedure, electrophysiological recordings, motor responses, and patient reactions to stimulation were continuously observed. Electrode placement was guided by MER patterns indicative of STN activity and motor responses, following Ben‐Gun orientation. The lead's middle contact points were positioned to align with the planned target, and the tip was oriented toward the substantia nigra. Depending on intraoperative findings, either directional or non‐directional electrodes were utilized. The devices used included Boston Scientific Gevia, Genus, and Medtronic Activa RC and PC models. The internal pulse generator was implanted in the midclavicular area on the same day.

Following electrode placement, postoperative 1 mm‐slice CT scans parallel to the AC‐PC line were fused with preoperative MRI data using BrainLab Elements and Medtronic Stealth software. The coordinates of the electrode tip were subsequently recorded as the postoperative coordinates. Revision surgery was performed for cases where deviations exceeding 2 mm from the preoperative plans were detected during fusion (1 patient). Apart from this single case, electrode placements were successful, with no more than a 2 mm deviation between the postoperative electrode position and the planned target. For the patient requiring revision, postoperative CT confirmed the new electrode position, and the final data for this patient were assessed based on post‐revision results.

In the postoperative period, deep brain stimulators were typically activated within 3 to 7 days. Activation was carried out by neurology and neurosurgery specialists, following a 12‐h discontinuation of medication to allow for motor response assessment and stimulation parameter adjustment. After determining optimal medication and stimulation settings, patients were discharged. If intolerable side effects or a lack of efficacy were observed, lead revision was considered; however, no revisions were performed for these reasons among the study participants.

### 2.3 Imaging Analysis

The preoperative and postoperative imaging data were retrospectively analyzed. MRI and CT slices were acquired at 1 mm. Patients' preoperative and postoperative electrode coordinates were calculated using BrainLab software, with the AC‐PC line determined by referencing the area just posterior to the anterior commissure and just anterior to the posterior commissure. The postoperative electrode position was identified through a lead detection algorithm to confirm accurate placement. The postoperative coordinate was accepted as the lead tip position. This evaluation was used solely to compare with the planning coordinate.

All MRI scans were performed using either a Philips MRI Ingenia 3.0 Tesla (Philips/Netherlands) or a Siemens Magnetom Symphony 1.5 Tesla (Siemens, Erlangen, Germany) scanner. The following parameters were used for each scanner to ensure high‐resolution structural imaging: For the 3 T MRI, a 3D magnetization‐prepared rapid acquisition gradient echo (MPRAGE) T1‐weighted sequence was acquired with a sagittal section thickness of 1.0 mm (no gap), TR of 2300 ms, TE of 2.98 ms, flip angle of 9°, FOV of 240 × 256 mm, matrix size of 240 × 256, TI of 900 ms, and voxel size of 1 × 1 × 1 mm^3^. The image plane was aligned sagittally along the hemispheric fissure and axially along the anterior/posterior commissure plane. For the 1.5 T MRI, axial high‐resolution contiguous 3D T1‐weighted images were obtained using an MPRAGE sequence with TR of 2500 ms, TE of 3.7 ms, TI of 730 ms, flip angle of 15°, section thickness of 1 mm, FOV of 256 mm, and matrix size of 256 × 256.

The volumes of Peripheral Gray Matter (cerebral‐cortical gray matter), Gray Matter, White Matter, Total Brain, Total Ventricle, Lateral Ventricle, and each STN were calculated using FSL (FMRIB Software Library). To standardize imaging data across different MRI devices (1.5 T vs. 3 T) and to ensure accurate measurements, all scans were first converted from DICOM to NIfTI format using dcm2niiX v1.0.2. Volumetric measurements were then processed using FSL SIENAX, which normalizes brain tissue segmentation and accounts for scanner‐specific intensity variations. The Brain Extraction Tool (BET) was applied to ensure uniform segmentation. To further standardize the images, all scans were normalized to a common space (MNI152_T1_1mm standard space), ensuring consistency across different scanners. Manual quality control was performed for all segmentations, and any identified errors were corrected through reprocessing to maintain accuracy in volumetric assessments. Only data meeting quality control standards were included in the study, and patients with unreliable data were excluded. Studies indicate that this method detects atrophy with an error rate of less than 1%, ensuring robust volumetric measurements [13]. Evans Index was calculated as the ratio of the anterior horn of the lateral ventricles to the maximum parietal diameter.

To grade brain atrophy, two scales commonly used in dementia assessment were applied. The Scheltens MTA Scale was used to rate medial temporal atrophy (scored from 0 to 4). The Pasquier Frontal GCA‐F Scale evaluated frontal cortical atrophy (scored from 0 to 3) [14].

### 2.4 Statistical Methods

All statistical analyses were performed using IBM SPSS Statistics (version 25). Descriptive statistics were calculated for all variables, including mean, standard deviation, minimum, and maximum values for continuous variables. Categorical variables were presented as percentage distributions, with classifications expressed in frequency (*n*) or percentage (%).

Pearson correlation analysis was used to assess linear relationships between variables. The correlation coefficient (*r*) was employed to evaluate the strength and direction of the relationship between two variables, with values ranging from −1 to +1. Values close to zero indicated a weak relationship, while values approaching −1 or +1 indicated a strong relationship.

To assess the relationship between STN coordinate shifts and anatomical parameters, we conducted both linear regression and multivariate regression analyzes. Linear regression analysis was used to evaluate the individual effects of independent variables on STN coordinate shifts. Multivariate regression analysis was performed to control for potential confounding factors and to assess the independent contributions of each variable while accounting for interdependencies between predictors. Statistical significance was determined at *p* < 0.05, with 95% confidence intervals reported for all analyzes. Multiple comparison corrections were not applied because each independent variable was assessed within a structured regression model, inherently controlling for false‐positive inflation.

STN coordinate shifts on the *X*, *Y*, and *Z* axes were analyzed in relation to volumetric and categorical variables. Each STN coordinate shift was treated as an independent variable, and absolute shifts from the zero point were used.

For categorical variables, data from the Scheltens MTA Scale and Pasquier Frontal GCA‐F Scale were applied. Outliers were identified using *z*‐scores, where values exceeding ±3 standard deviations were considered outliers. These outliers were evaluated for their impact on the statistical models and excluded only if they significantly distorted the results. This approach ensured the reliability of the findings while preserving the integrity of biologically meaningful data. No adjustments were made for categorical variables.

## 3 Results

A total of 101 participants were included in this study, comprising 49 females and 52 males. All participants underwent bilateral STN‐DBS surgery due to IPD, resulting in a total of 202 STNs analyzed across both hemispheres. The mean age of participants was calculated as 57.75 years (range: 35–75). Brain volume measurements showed a mean total brain volume of 1419.67 cm^3^, peripheral gray matter volume of 809.25 cm^3^, gray matter volume of 1090.71 cm^3^, and white matter volume of 328.65 cm^3^. These values and the distributions of other measurements are detailed in Table 1.

**TABLE 1:** Demographic and clinical characteristics.

Variable	Variable overview				
	**Male**	**Female**	**Total**		
Participants (*n*)	52	49	101		
	**Mean**	**Std Dev**	**Min**	**Max**	
Age	57.75	7.87	35	75	
Peripheral gray matter volume (cm^3^)	809.25	118.59	515.28	1040.36	
Total ventricle volume (cm3)	59.47	19.58	27.41	101.35	
Gray matter volume (cm3)	1090.71	200.99	862.42	1415.32	
White matter volume (cm3)	328.65	203.29	165.23	801.97	
Total brain volume (cm3)	1419.67	78.93	1202.55	1599.42	
Evans index ratio	0.256	0.034	0.19	0.356	
Lateral ventricle volume (cm3)	29.23	10.28	15.30	78.80	
Right STN volume (cm3)	0.104	0.019	0.07	0.21	
Left STN volume (cm3)	0.104	0.017	0.07	0.15	
AC‐PC length (mm)	24.33	1.79	19.90	28.60	
	**Grade 0**	**Grade 1**	**Grade 2**	**Grade 3**	**Grade 4**
Scheltens MTA scale (%)	52.48%	31.68%	12.87%	1.98%	0.99%
Pasquier frontal GCA‐F scale (%)	35.64%	41.58%	21.78%	0.99%	—
	**Center**	**Medial**	**Anterior**	**Lateral**	**Posterior**
Final lead ben‐gun orientation (*n*)	154	23	15	5	5
	***X***	***Y***	**Z**		
STN coordinates—mm (mean)					
Preoperative planning					
Left	−12.23	−2.01	−4.09		
Right	12.48	−1.89	−4.09		
Total	±12.36	−1.95	−4.09		
Postoperative lead tip					
Left	−10.92	−4.30	−6.96		
Right	11.06	−4.02	−6.74		
Total	±10.99	−4.16	−6.85		

*Note:* This table provides an overview of the distribution of demographic characteristics, brain volumes, atrophy scores, planning and postoperative coordinates, and lead tip positions according to the selected Ben‐Gun orientation.

**FIGURE 1:**
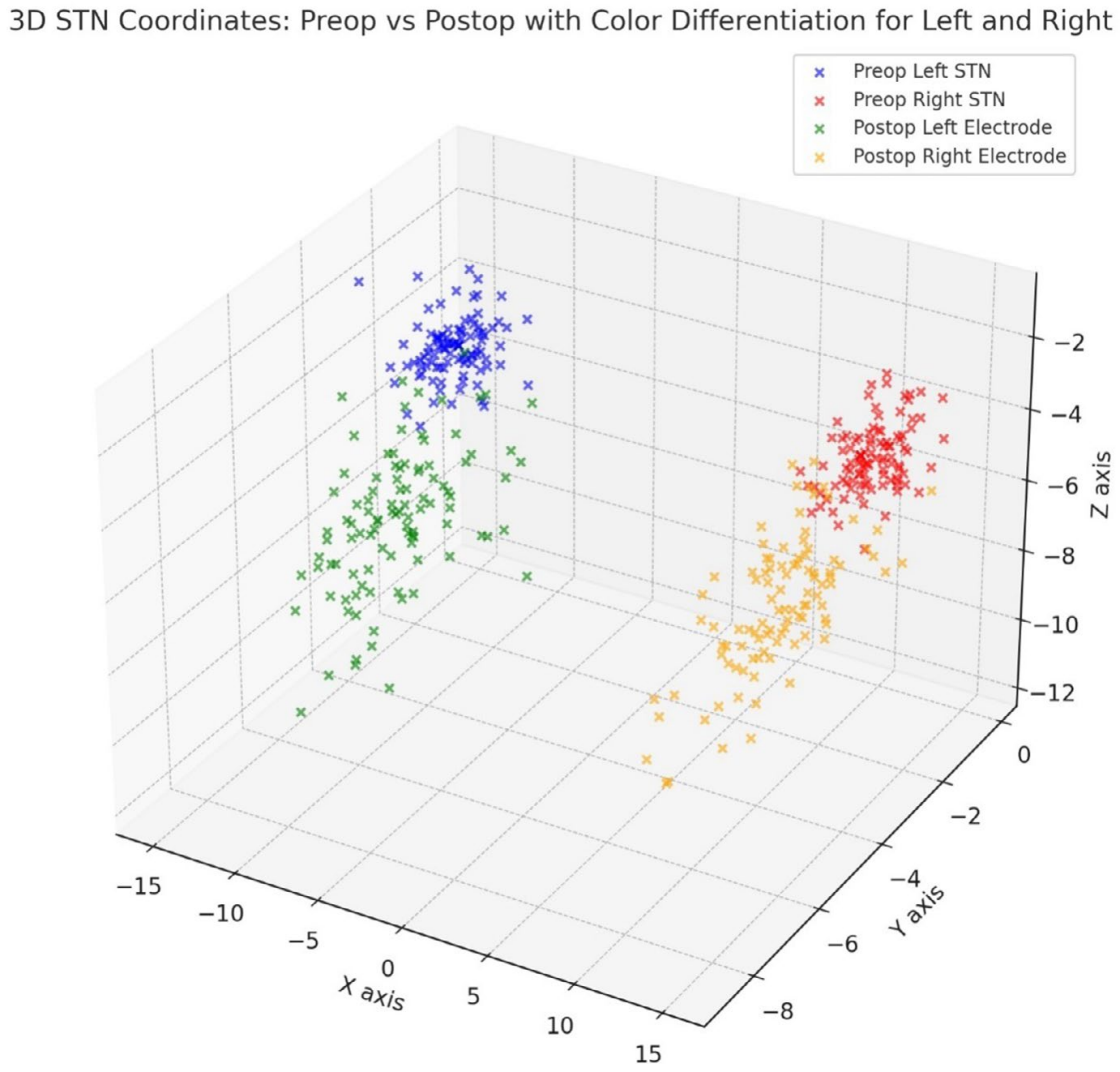
Distribution of Subthalamic Nucleus (STN) Planning Target Coordinates and Postoperative Lead Tip Coordinates in 3D Axes. This figure provides a 3D visualization of the discrepancies between planned and postoperative STN coordinates, illustrating the shifts along all axes.

The mean preoperative STN coordinates were determined to be ±12.36 mm on the *X*‐axis, −1.95 mm on the *Y*‐axis, and −4.09 mm on the *Z*‐axis. Postoperative averages were recorded as ±10.99 mm on the *X*‐axis, −4.16 mm on the *Y*‐axis, and − 6.85 mm on the *Z*‐axis. Final lead orientations were “center” in 76.2% of participants and “other” in 23.8% (Table 1, Figure 1).

In terms of relationships, a statistically significant positive correlation was found between age and STN *X* coordinate shift (*r* = 0.35, *p* < 0.001). Each additional year of age resulted in an approximate lateral shift of 0.046 mm on the STN *X*‐axis. This finding suggests a slight lateral displacement of the STN on the *X*‐axis as age progresses.

Correlation analyses between brain volumes and STN coordinates yielded several significant findings. A statistically significant positive correlation was observed between Peripheral Gray Matter Volume and STN *X* coordinate shift (*r* = 0.16, *p* = 0.027). For each 1 cm^3^ reduction in cortical gray matter volume, a medial shift of approximately 0.0014 mm was observed on the STN *X*‐axis. Additionally, a significant negative correlation was identified between White Matter Volume and STN *X* coordinate shift (*r* = −0.14, *p* = 0.044), indicating that each 1 cm^3^ decrease in white matter volume was associated with a lateral coordinate shift of approximately 0.0007 mm on the STN *X*‐axis. However, no significant correlations were found between Gray Matter Volume, Total Brain Volume, STN volume, and STN coordinate shift.

Examining ventricle volumes, statistically significant positive correlations were found between Total Ventricle Volume and STN *X* and *Z* coordinate shifts (*X*: *r* = 0.42, *p* < 0.001; *Z*: *r* = 0.22, *p* = 0.001). Each 1 cm^3^ increase in total ventricle volume caused an STN coordinate shift of 0.0227 mm on the *X*‐axis and 0.0087 mm on the *Z*‐axis. Similarly, positive correlations were detected between Lateral Ventricle Volume and STN *X* and *Z* coordinate shifts (*X*: *r* = 0.47, *p* < 0.001; *Z*: *r* = 0.19, *p* = 0.007). Each 1 cm^3^ increase in lateral ventricle volume resulted in an STN shift of approximately 0.0484 mm on the *X*‐axis and 0.0142 mm on the *Z*‐axis. The Evans Index Ratio showed a significant lateral coordinate shift on the STN *X*‐axis (*r* = 0.27, *p* < 0.001), with each 0.1 increase in the Evans Index leading to a 0.845 mm shift on the STN *X*‐axis. However, no significant changes were observed on the *Y* and *Z* axes.

**FIGURE 2:**
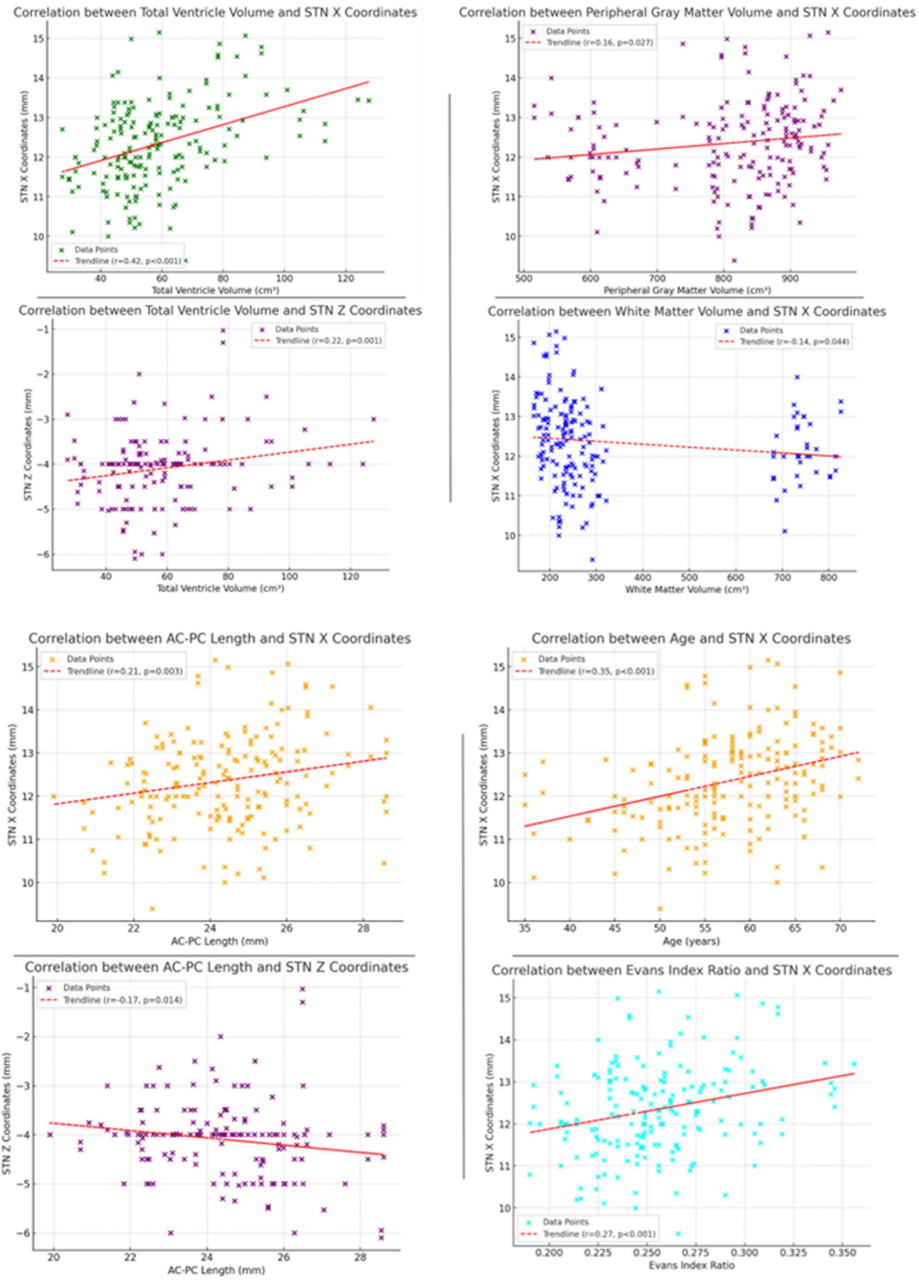
Display of Correlation Curves for Significant Variables. This figure illustrates the effects of significant demographic and clinical variables on the subthalamic nucleus (STN) in Parkinson's patients using correlation curves. Each graph shows the relationship between significant variables and STN shifts along the axes, depicted through linear regression curves based on multivariate analyses.

Significant correlations were also found between AC‐PC length and STN *X* and *Z* coordinate shifts (*X*: *r* = 0.21, *p* = 0.003; *Z*: *r* = −0.17, *p* = 0.014). Each 1 mm increase in AC‐PC length resulted in a 0.124 mm positive shift on the STN *X*‐axis and a negative shift of 0.074 mm on the *Z*‐axis. This finding suggests that AC‐PC length may influence the lateral and vertical shift of the STN relative to the surgical target (Table 2, Figure 2).

**TABLE 2:** Correlation coefficients and shift values for significant variables across the *X*, *Y*, and *Z* Axes of the STN.

Variable	STN *X*			STN *Y*		STN *Z*		
	*r*	*p*	Shift (mm)*	*r*	*p*	*r*	*p*	Shift (mm)*
Age	0.35	**< 0.001**	0.046	−0.12	0.091	0.13	0.07	
Peripheral gray matter volume	0.16	**0.027**	0.0014	−0.051	0.47	−0.007	0.92	
Gray matter volume	0.13	0.064		−0.059	0.404	−0.014	0.844	
White matter volume	−0.14	**0.044**	−0.0007	0.079	0.262	−0.001	0.985	
Total brain volume	−0.075	0,29		0.076	0.282	−0.033	0.636	
Total ventricle volume	0.42	**< 0.001**	0.0227	−0.037	0.604	0.22	**0.001**	0.0087
Lateral ventricle volume	0.47	**< 0.001**	0.0484	−0.016	0.826	0.19	**0.007**	0.0142
Evans index ratio	0.27	**< 0.001**	0.845	−0.074	0.296	0.063	0.373	
STN volume	0.13	0.067		0.024	0.739	−0.081	0.255	
AC‐PC length	0.21	**0.003**	0.124	0.015	0.834	−0.17	**0.014**	−0.074
Scheltens MTA scale		**< 0.001**			0.965		0.327	
Pasquier frontal GCA‐F scale		**0.003**			0.326		0.139	

*Note:* This table presents the correlation coefficients (*r*) and *p*‐values for associations between demographic, clinical, and anatomical variables and STN coordinate shifts across the *X*, *Y*, and *Z* axes. Variables with no significant results are excluded from the respective axes. The bold values indicate statistically significant data. (*) STN shifts are reported in millimeters relative to changes in different variables. Age‐related shifts are expressed per year, volumetric shifts (e.g., gray matter volume, white matter volume, total and lateral ventricle volumes) per cm^3^, ratio‐based shifts (Evans Index) per 0.1 unit, and AC‐PC length shifts per mm. Each value represents the expected displacement of the STN per unit increase in the corresponding variable.

**FIGURE 3:**
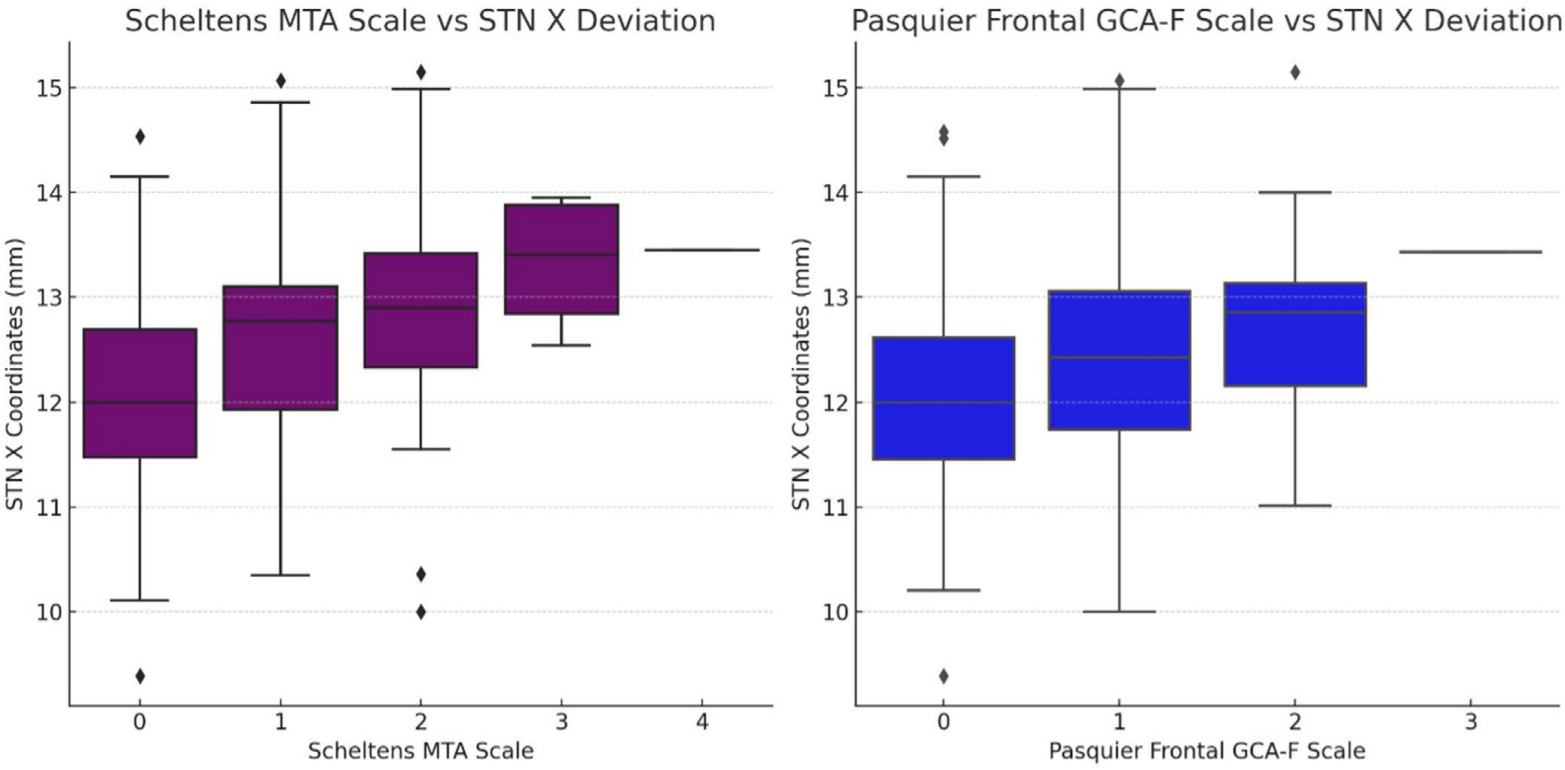
Distribution of Atrophy Assessment Scales and STN *X*‐Axis Shifts. This figure illustrates the relationship between the Scheltens MTA and Pasquier Frontal GCA‐F atrophy assessment scales and shifts along the *X*‐axis. The effect of structural brain changes on STN target coordinate shift.

Regarding atrophic grading scales, significant positive correlations were observed between STN *X* shift and the Scheltens MTA Scale and Pasquier Frontal GCA‐F Scale. These results indicate that higher atrophy scores are associated with the lateral shift of the STN on the *X*‐axis. However, no significant shifts were detected on the *Y* and *Z* axes. These findings suggest that various atrophic grading systems may contribute to the lateral displacement of the STN on the *X*‐axis (Figure 3).

## 4 Discussion

This study comprehensively examined the relationship between shifts in targeted STN coordinates on the *x*, *y*, and *z* axes and various anatomical parameters, such as brain volume measurements and ventricle widths, in Parkinson's patients undergoing STN‐DBS surgery. Our findings indicate that significant volumetric changes in brain structure may influence STN positioning, with increases in certain parameters causing shifts on the *x* and *z* axes. Notably, age, peripheral gray matter and white matter volumes, ventricle volume, Evans index, AC‐PC length, and atrophy scales were found to lead to significant shifts in STN coordinates. Additionally, the extent of STN shift in millimeters due to changes in these clinical features is demonstrated.

Although advancements in imaging technologies have significantly reduced the reliance on indirect targeting, certain cases still require its use when direct targeting proves insufficient [15]. One of the primary reasons for this is the suboptimal quality of MRI images. Factors such as the unavailability of high‐resolution imaging devices in all clinical settings, the lack of experience among MRI technicians, contour distortion in T2 images, and motion artifacts from tremor can impair MRI quality [16]. In such cases, the STN may not be fully visualized, making indirect coordinates a commonly employed validation tool [17]. In our practice, we continue to use indirect targeting coordinates as a confirmation tool, even when direct targeting with 3 T MRI and atlas‐based segmentation is employed. Our study has provided clinicians with valuable data to guide decision‐making when using indirect targeting coordinates.

Stereotactic atlas coordinates provide clinicians with the average coordinates of many targets, but these averages often carry error margins that exceed acceptable limits [18]. This discrepancy is associated with the variability of individual characteristics. In our study, we found a statistically significant positive relationship between age and lateral shift of the STN on the *X*‐axis, independent of other variables. This finding aligns closely with Pereira et al. [19] who reported that age is linked to lateral shift, independent of ventricle width. Additionally, we demonstrated that each additional year of age results in approximately 0.046 mm of lateral shift. Other studies in the literature also support the association between age and STN lateral shift [20, 21]. Age‐related atrophic changes in the internal anatomy of the STN or surrounding structures may account for this shift [22]. Moreover, ventricle enlargement can also lead to shifts in surgical targeting. Pereira et al. demonstrated that third ventricle enlargement contributes to lateral displacement of the STN [19]. Similarly, Keuken et al. [20] identified third ventricle width as a primary predictor of STN lateralization. This lateralization may also be associated with reduced white matter volume [23]. Our study evaluated the relationship between total and lateral ventricle volumes, as well as white matter volume, and the positioning of the STN on each of the three axes. We demonstrated that an increase in ventricle volume or a decrease in white matter volume independently contributes to STN lateralization, regardless of other variables. This finding serves to complement previous studies.

The association between increased peripheral (cerebral‐cortical) gray matter volume and more lateral STN localization is noteworthy. Since our study conducted spot measurements rather than longitudinal analysis, it is difficult to determine whether this is a structural characteristic or if atrophy in peripheral gray matter causes medial migration of the STN. However, according to Jia et al. [24] certain cortical gray matter volumes are higher in Parkinson's patients compared to healthy individuals within the neurodegenerative process. On the other hand, other studies have reported reduced white matter volume in Parkinson's patients, suggesting that neurodegeneration may lead to an increase in gray matter and a decrease in white matter [25]. Ventriculomegaly, which may be aggravated by aging and other factors, is also a marker of neurodegenerative processes [26]. Rather than focusing on absolute volumetric changes due to disease progression, our study highlights how these variations influence the spatial positioning of the STN. This perspective is particularly relevant for DBS planning, where anatomical variability must be considered to optimize targeting accuracy. Our study demonstrates that reduced white matter volume and increased ventricle and cortical gray matter volumes are associated with lateral localization of the STN. In this context, lateral STN migration could be part of the neurodegenerative process. This finding is significant both for surgical planning and as a potential factor in postoperative complications in DBS surgery. Previous case reports on STN and VIM‐DBS indicate that brain atrophy can result in lateral displacement of DBS electrodes along with brain tissue, a factor that clinicians should be mindful of [22, 27].

Patient‐specific planning enhances the effectiveness of DBS and reduces the risk of side effects [11, 28, 29]. Over the past decade, new‐generation software that integrates atlas‐based automatic segmentation has become available, allowing the alignment of individual anatomy with atlas space to define patient‐specific anatomical boundaries [30, 31]. These programs use methods like diffeomorphic registration algorithms or tissue probability maps [32]. Our study provides information to aid in patient‐specific targeting by calculating the degree of STN shift associated with certain volumetric changes (Table 2). Future large‐scale studies and data collected from healthy individuals could help improve automatic segmentation systems by generating more accurate maps through volumetric analysis.

The AC‐PC distance is a critical reference point in stereotactic surgery [33]. Stereotactic coordinates are calculated by considering the midpoint of this line as zero. Traditional indirect targeting attempts to locate the STN using AC‐PC‐based diagrams. Our study found that an increase in AC‐PC length results in shifts along the *x* and *z* axes, suggesting that AC‐PC diagrams may have a higher margin of error for STN positioning. This finding aligns with the study by Caire et al. [34] which supports this perspective. Additionally, the literature suggests that targeting based on the red nucleus may be more reliable [9].

To provide clinicians with a more practical approach beyond volumetric measurements, we included two dementia scales—Scheltens Medial Temporal Atrophy and Pasquier Frontal GCA‐F—that are not specifically related to Idiopathic Parkinson's Disease [35, 36]. We identified significant correlations between these scales and lateral shift on the *x*‐axis. Notably, in the Scheltens scale, as the grade increased, lateral displacement also increased. These visual scales are two of the most widely applied tools in dementia evaluation [14]. Unfortunately, no alternative visual criteria were available specifically for Parkinson's‐related atrophy. Clinicians familiar with these atrophy scales and their imaging patterns may find them useful for recognizing cases in which the STN is positioned more laterally.

The use of intraoperative methods such as macrostimulation and microelectrode recording (MER) remains a topic of ongoing debate [37, 38]. While some researchers view the use of these techniques as unnecessary, we continue to emphasize their importance. As shown in Figure 1, there were differences between the target determined during the planning phase and the final lead position established based on MER and macrostimulation data. We believe, as highlighted in our study, that these differences may arise from individual anatomic and physiological variations. Even when the anatomically targeted location seems accurate, we believe that electrophysiological and clinical responses should be considered; these responses should be prioritized when assessing target accuracy. Furthermore, our study suggests that advancing direct targeting methods, with the potential to increase accuracy, could guide future research toward clinically and electrophysiologically precise direct targeting techniques.

In conclusion, anatomical factors such as cortical gray matter and white matter volumes, ventricle volume, Evans index, and AC‐PC length play a significant role in influencing STN electrode placement. Additionally, visual atrophy grading provides valuable insights into STN lateralization. This study suggests that accounting for these anatomical variations in surgical planning could improve electrode placement accuracy and treatment outcomes. Furthermore, our findings may contribute to more precise targeting in new‐generation direct targeting methods. By demonstrating the influence of anatomical variability on STN localization, this study underscores the need for further research on optimizing indirect targeting strategies, improving volumetric assessment techniques, and refining DBS planning to enhance clinical outcomes.

This study has several limitations. As a retrospective, single‐center study, data were collected from past patient records, possibly lacking specific details more accessible in a prospective study. These data limitations and the specific patient population restrict generalizability, requiring confirmation through multicenter studies. A statistical limitation of this study is that multiple comparison corrections were not applied, and statistical significance was determined at *p* < 0.05. However, multivariate regression analysis was employed to control for potential confounding effects and reduce the likelihood of false positives. Brain volume and atrophy assessments relied on MRI data, where variability in image quality, equipment differences, or subjective scoring may have influenced the results, particularly in atrophy evaluations. Additionally, this study did not directly assess the effects of STN lateralization on surgical success or patient outcomes. Future studies exploring the clinical implications of STN positioning shifts could significantly enhance the applicability and relevance of this research. Despite these limitations, this study addresses an important knowledge gap in the field.

## Ethics Statement

We hereby state that the study was initiated with the approval of the local ethics committee under approval number 2024‐46.

## Consent

We confirm that we have read the Journal's position on issues involved in ethical publication and affirm that this work is consistent with those guidelines. Written informed consent was obtained from all participants.

## Conflicts of Interest

The authors declare no conflicts of interest.

## Data Availability

The data that support the findings of this study are available from the corresponding author (OH) upon reasonable request.
